# Influence of maternal nutrition and one-carbon metabolites supplementation on bovine antimicrobial peptides in fetal and maternal tissues

**DOI:** 10.3389/fvets.2024.1505427

**Published:** 2024-12-10

**Authors:** Mojtaba Daneshi, Pawel P. Borowicz, Mara R. Hirchert, Yssi L. Entzie, Jessica G. Syring, Layla E. King, Kazi Sarjana Safain, Muhammad Anas, Lawrence P. Reynolds, Alison K. Ward, Carl R. Dahlen, Matthew S. Crouse, Joel S. Caton

**Affiliations:** ^1^Department of Animal Sciences, Center for Nutrition and Pregnancy, North Dakota State University, Fargo, ND, United States; ^2^Department of Agriculture and Natural Resources, University of Minnesota Crookston, Crookston, MN, United States; ^3^Department of Veterinary Biomedical Sciences, University of Saskatchewan, Saskatoon, SK, Canada; ^4^United States Department of Agriculture, Agriculture Research Service, U.S. Meat Animal Research Center, Clay Center, NE, United States

**Keywords:** β-defensins, cathelicidins, developmental programming, early pregnancy, epigenetic modifications, innate immunity, nutrition restriction, one-carbon metabolism

## Abstract

**Introduction:**

Maternal nutrition during pregnancy critically influences offspring development and immune function. One-carbon metabolites (OCM) are epigenetic modifiers that may modulate antimicrobial peptide (AMP) expression, which is vital for innate immunity. This study investigated the effects of maternal nutrient restriction and OCM supplementation on mRNA expression of AMP in fetal and maternal lung, mammary gland, and small intestine of beef cattle.

**Methods:**

Twenty-nine crossbred Angus beef heifers were synchronized for estrus and artificially inseminated. They were assigned to one of four treatments in a 2 × 2 factorial design: nutritional plane [control (CON) vs. restricted (RES)] and OCM supplementation [without OCM (−OCM) or with OCM (+OCM)]. Heifers on the CON diet were fed to gain 0.45 kg/day, while RES heifers were fed to lose 0.23 kg/day. Treatments were applied from day 0 to 63 of gestation, after which all heifers were fed a common diet to gain 0.45 kg/day until day 161 of gestation, when samples were collected. Quantitative RT-qPCR was used to assess mRNA expression of AMP.

**Results:**

Nutritional plane had no effect (*p* ≥ 0.24) on mRNA expression of AMP in either the fetus or dams. However, the mRNA expression of cathelicidin5 (*CATHL5*; *p* = 0.07) and bovine neutrophil β-defensin5 (BNBD5; *p* = 0.07) in the fetal lung and mammary gland, respectively, was lower in the +OCM groups compared to the −OCM groups. In the maternal small intestine, the expression of enteric β-defensin (EBD) was lower (*p* = 0.01) in the +OCM groups compared to the −OCM groups. Additionally, in the maternal lung, there was a tendency (*p* = 0.06) for an interaction in *CATHL5* mRNA expression, with the RES + OCM group showing greater expression compared to the CON + OCM (*p* = 0.07) and RES − OCM (*p* = 0.08) groups.

**Discussion:**

Our findings suggest that while restricted maternal nutrition did not affect mRNA expression of AMP, OCM supplementation modulated AMP expression in both fetal and maternal tissues. Further research is needed to elucidate the mechanisms underlying OCM’s impact on AMP expression.

## 1 Introduction

Maternal nutrition during pregnancy plays a crucial role in influencing the health and development of offspring (1, 2). Factors such as drought-induced forage shortages and inadequate intake of essential minerals and supplements can disrupt maternal nutrition, leading to lasting effects on fetal growth, immune function, and metabolic regulation in livestock (3, 4). This phenomenon, known as developmental programming, suggests that stressors, including nutritional stress, during critical developmental windows can elicit both immediate and prolonged consequences in the offspring (5). A principal mechanism by which maternal nutrition can affect fetal development is through epigenetic modifications (6–8).

Central to this epigenetic control are one-carbon metabolites (OCM), which include methyl-donors such as folate, vitamin B_12_, methionine, and choline. These metabolites serve as vital epigenetic modifiers, influencing DNA methylation and regulating the expression of genes involved in various biological processes, including immune function (9–12). Dysregulation of these metabolites and the one-carbon metabolism pathway can lead to immune dysfunction (13, 14). Studies indicate increased maternal plasma concentrations of OCM are associated with greater expression of genes related to the immune system, histone modification, and RNA processing in offspring (15). Additionally, our previous research has shown that nutrient restriction in pregnant beef heifers can result in reduced concentrations of methionine, an essential OCM, in allantoic fluid and increased concentrations of homocysteine in maternal serum during early gestation (16). These alterations in OCM levels can subsequently alter metabolic pathways related to one-carbon metabolism and influence epigenetic modifications (10). This highlights the potential impact that altering OCM supply could have on the developing immune system.

Antimicrobial peptides (AMP), such as β-defensins (17), cathelicidins (18), and S100 proteins (19), are diverse molecules that serve as a first-line host innate defense mechanism. Typically short and positively charged, AMP interact with the negatively charged membranes of microbes, disrupting the membrane and causing microbial death (20, 21). They can also target other microbial components, such as DNA or proteins (22). AMP offers several advantages over traditional antibiotics. They are less likely to trigger resistance in bacteria, act much faster, and can work against a wider range of bacteria, fungi, and enveloped viruses. This makes them particularly effective against even those strains of bacteria that are resistant to multiple antibiotics (23, 24). AMP exhibit potent bactericidal activity against pathogens affecting the respiratory tract, intestine, and udder, such as *Mannheimia haemolytica*, *Histophilus somni*, *Pasteurella multocida*, *Escherichia coli*, and *Staphylococcus aureus* (25–29). Epigenetic pathways, including DNA methylation and histone modification, play key roles in regulating the expression and production of AMP (30, 31). Bovine respiratory disease (32), infectious diarrhea (33), and mastitis (34) are significant health concerns in cattle industry, contributing to substantial economic losses and health complications. The rise in antimicrobial resistance due to the overuse of synthetic antimicrobials further emphasizes the need to enhance the natural immune system to prevent diseases and combat pathogens (35, 36). Therefore, understanding the variables that affect the epigenetic control of AMP is essential for enhancing their expression and boosting the natural immunity of cattle. However, the influence of maternal nutrition and strategic OCM supplementation on AMP expression remains to be elucidated.

The objective of this study was to determine the effects of restricted maternal nutrition during early gestation, with or without OCM supplementation, on mRNA expression of AMP in the bovine fetal and maternal lung, mammary gland, and small intestine. We hypothesized that maternal nutrient restriction would reduce antimicrobial peptide expression, while one-carbon supplementation would at least partially ameliorate these effects. The data will provide insight into how the maternal nutritional level affects the innate immune system in the bovine fetus and dams, and whether strategic supplementation with methyl-donor molecules may support normal innate immunity maturation under conditions of poor maternal nutrition.

## 2 Materials and methods

### 2.1 Animal ethics

All methodologies and experiments adhered to relevant guidelines and regulations. The design of the experiment, the management of animals, and the tissue collection processes were approved by the Institutional Animal Care and Use Committee at North Dakota State University (IACUC #20220059) on October 26, 2022.

### 2.2 Animals, diet, and treatment

Seventy-two Angus heifers were transported from the Central Grasslands Research Extension Center (Streeter, ND, USA) to the Animal Nutrition and Physiology Center at North Dakota State University (Fargo, ND, USA). After a 14-day adaptation to the feeding system, heifers were subjected to a 7-day Select Synch + CIDR estrus synchronization protocol (37) and artificially inseminated 18 to 22 h following the detection of estrus. To explore the effects of maternal nutrition and OCM on fetal development during gestation while removing sex-specific effects (38, 39), only female-sexed semen from a specific bull (Connealy Maternal Made [ST Genetics, Navasota, TX, USA]) was used for insemination. Of these 72 heifers, 29 (~ 14 months of age) successfully conceived [CON − OCM (*n* = 7), CON + OCM (*n* = 8), RES − OCM (*n* = 7), and RES + OCM (*n* = 7)]. The average initial body weight was 436 ± 42 kg. Upon breeding, the heifers were allocated into four different dietary groups based on a 2 × 2 factorial design that considered the level of nutritional plane (control vs. restricted) and OCM supplementation (with or without OCM). The heifers were individually fed daily in an electronic head gate facility (American Calan; Northwood, NH) at 0800 h daily.

Heifers on the control (CON) diet were fed to achieve 100% of the National Academy of Sciences, Engineering, and Medicine (NASEM) (40) nutritional requirements aiming for an average daily gain (ADG) of 0.45 kg/day, though they actually gained 0.60 kg/day. This regimen intended to bring them to 80% of their mature body weight by calving. Conversely, heifers on the restricted (RES) intake regimen were fed less to achieve a weight loss of 0.23 kg/day, mimicking the natural production responses observed in heifers undergoing dietary and environmental changes in early gestation (40). Their diets consisted of a total mixed ration including corn silage, alfalfa hay, corn grain, mixed alfalfa/grass hay, and a vitamin/mineral premix (Trouw dairy VTM w/Optimins, Trouw Nutrition USA, Highland, IL, USA). Diet compositions are more fully described in (41). Diets were formulated based on initial body weight at breeding to contain 2.25 Mcal/kg ME, 9.75% CP, and 58.6% NDF. Heifers were weighed weekly, and their individual feed intake was adjusted throughout the study to achieve the targeted body weight gains. Heifers supplemented with OCM (+OCM) received daily doses of 7.4 g of rumen-protected methionine (Smartamine, Adisseo, Beijing, China) and 44.4 g of rumen-protected choline (ReaShure, Balchem Inc., New Hampton, NY, USA) in a corn carrier, following dosages from previous studies (42, 43). They also received weekly intramuscular injections of vitamin B_12_ (50 mg of cyanocobalamin/mL; MWI Animal Health, Boise, ID, USA) and folate (53.33 mg of folic acid/mL; Spectrum Chemical Mfg. Corp., New Brunswick, NJ, USA) supplements, aimed at delivering 320 mg of folic acid and 20 mg of vitamin B_12_ each week, following previously described methods (43, 44). In contrast, the non-OCM supplemented heifers (−OCM) were given only the corn carrier daily and received weekly saline injections intramuscularly. These treatment protocols continued until day 63 of gestation, after which all heifers were managed on the CON − OCM treatment targeting a daily gain of 0.45 kg for the remainder of the study.

Pregnancy confirmation occurred on day 35 via transrectal ultrasonography by detecting fetal heartbeats, and a subsequent ultrasound on day 63 assessed fetal sex (45), retaining only pregnant heifers with female fetuses for further study stages.

### 2.3 Sample collection and preparation

Heifers were slaughtered on day 161 of gestation. The gravid uterus was promptly removed, and the fetus was immediately separated from the placenta, and subsequently dissected. Given that the cranioventral area of the lung is predominantly affected in bovine respiratory disease (46), lung samples from both the fetus and the dam were collected from this region (left cranioventral). Reflecting findings that high levels of AMP mRNA are present in the ileum (47), samples from this region were obtained from a section 10 cm proximal to the ileocecal junction. Additionally, considering the involvement of the mammary gland parenchyma in mastitis (48) and the uniform expression of AMP across different quarters (17), mammary gland samples were taken from the parenchyma of the right forequarters. All samples were wrapped in aluminum foil, snap-frozen in liquid nitrogen, and stored at −80°C until further analysis.

### 2.4 RNA extraction

Total RNA was extracted using the RNeasy® Plus Universal Kit (Qiagen, Germantown, MA, USA), following the manufacturer’s protocol and tissues were lysed using the TissueLyser LT (Qiagen Germantown, MA, USA). The RNA concentration was determined using the Qubit® RNA BR Assay Kit (Thermo Fisher Scientific, MA, USA) and measuring it with a Qubit 3.0 Fluorometer (LifeTechnologies, Carlsbad, CA, USA).

### 2.5 cDNA synthesis

The RNA was reverse transcribed into cDNA utilizing the High-Capacity Reverse Transcription Kit (Thermo Fisher Scientific, MA, USA) following the manufacturer’s protocol. The final cDNA concentration was 100 ng/μl in a volume of 20 μl, and samples were stored at −20°C until use.

### 2.6 RT-qPCR

The target AMP genes were as follows: lingual antimicrobial peptide (*LAP*), tracheal antimicrobial peptides (*TAP*), enteric β-defensin (*EBD*), bovine neutrophil β-defensin4 (*BNBD4*), bovine neutrophil β-defensin5 (*BNBD5*), S100 calcium binding protein A7 (*S100A7*), and cathelicidin5 (*CATHL5*). For each gene, the primers were designed using NCBI/Primer-BLAST or sourced from previous literature.

Optimization was conducted to determine the optimum cDNA concentration and primer efficiencies in each tissue type (49). The amplification efficiency (*E*) of target genes was evaluated by plotting the cycle threshold (Ct) versus *log* concentration. The *E* was calculated using the equation $E\;\left(\%\right)=\left[10^{-1/\mathrm{slope}}-1\right]\times 100$ , with acceptable efficiencies ranging between 90 and 110% (50, 51). Details of the primer sequences used in the qPCR analysis can be found in Table 1. The primer sets for two genes (*S100A7* and *TAP*) fell outside the acceptable efficiency range, likely due to low expression levels, as they exhibited high Ct values at the highest cDNA concentrations. Consequently, the relative mRNA expression of *S100A7* and *TAP* were deemed below our limit of detection and were not further evaluated in this study. All other primers demonstrated efficiency within the acceptable range (90–110%). The results of the amplification efficiency experiment, including slope, R^2^, and efficiency, are summarized in Supplementary Tables S1, S2.

**Table 1:** Primer sequences for validation and evaluation of amplification efficiency in RT-qPCR.

Gene^1^	Sequence (5′-3′)^2^	Product size, bp	Accession no.^3^	Tissues analyzed	Source
*LAP*	F: CCTGTCTGCTGGGTCAGGATTTA	134	NM_203435.4	Maternal small intestine	NCBI Primer-BLAST
	R: TTACTTGGGCTCCGAGACAGG				
	F: GCCAGCATGAGGCTCCATC	194		Maternal mammary gland	(85–87)
	R: CTCCTGCAGCATTTTACTTGGGCT				
*EBD*	F: TATAAAGCGGCAAGAGCAGCC	102	NM_175703.3	Maternal small intestine	(88, 89)
	R: AGCATTTTACTGAGGGCGTGA				
*BNBD4*	F: CACAGCCTGCACAGAATTCCTC	172	NM_174775.1	Fetal and maternal lung	NCBI Primer-BLAST
	R: ACTCTTTGAGTAAATCCTGACCCA				
*BNBD5*	F: CCTAGTCCTGTCTGCTGGGTC	122	NM_001130761.1	Fetal and maternal mammary gland	NCBI Primer-BLAST
	R: AGGTGCCAATCTGTCTCATGTTG				
*CATHL5*	F: ACCTCCCAAGGAGGACGATG	152	NM_174510.3	Fetal mammary gland	NCBI Primer-BLAST
	R: TGACTGTCCCCACACACTCT				
	F: TCGGGAGTAACTTCGACATCACCT	141		Fetal and maternal lung	(89, 90)
	R: GGCCCACAATTCACCCAATTCTGA				
*GAPDH*	F: CTGCCCGTTCGACAGATAGC	153	NM_001034034.2	All	NCBI Primer-BLAST
	R: GATGGCGACGATGTCCACTT				
*ACTB*	F: CCGCAAATGCTTCTAGGCGG	189	NM_173979.3	All	NCBI Primer-BLAST
	R: ACTGCTGTCACCTTCACCGT				
*HPRT1*	F: CAGTTGCTGCATTCCCGAAC	125	NM_001034035.2	All	NCBI Primer-BLAST
	R: TTCCAGTCAATAGTGGTGTGGT				

^1LAP = lingual antimicrobial peptide, EBD = enteric β-defensin, BNBD4 = bovine neutrophil β-defensin4, BNBD5 = bovine neutrophil β-defensin5, CATHL5 = cathelicidin5. GAPDH = Glyceraldehyde-3-phosphate dehydrogenase. ACTB = Actin beta. HPRT1 = Hypoxanthine-guanine phosphoribosyltransferase1. 2F = Forward; R = Reverse. 3Accession numbers from NCBI database http://www.ncbi.nlm.nih.gov.^

Glyceraldehyde-3-phosphate dehydrogenase (*GAPDH*), actin beta (*ACTB*) and hypoxanthine phosphoribosyltransferase 1 (*HPRT1*) were used as reference genes. We employed the *geNorm* tool to assess the stability of their expression across all tissue samples. The lower *M*-values calculated by *geNorm* indicate more stable expression (52), and in our study, reference genes were considered stable if their *M*-values were below 0.5 (53).

Quantitative real-time PCR was performed using a QuantStudio™ 3 System (Thermofisher Scientific, MA, USA), with a 10 μl reaction mixture comprising 2 μl of the diluted cDNA (10 ng), 5 μl of 2× SYBR Green Master Mix (Bio-Rad Laboratories, Hercules, CA), 0.5 μl each of 10 μM forward and reverse primers (final concentration of 500 nM), and 2 μl of nuclease-free water. The amplification protocol included an initial denaturation at 95°C for 20 s, followed by 40 cycles of denaturation at 95°C for 1 s and annealing/elongation at 60°C for 20 s. A dissociation melt curve was determined at the end of each run, involving a brief denaturation at 95°C for 1 s, annealing at 60°C for 20 s, and a final denaturation at 95°C for 1 s. All runs included a negative control (without cDNA) and were performed in triplicate on a 96-well reaction plate (Thermo Fisher Scientific, MA, USA). Relative gene expression levels were quantified using the 2^-ΔΔCT^ method (54), employing *GAPDH*, *ACTB*, and *HPRT1* as reference genes for normalization, and using the CON − OCM group as control (set to 1).

### 2.7 Statistical analysis

Data were analyzed using the PROC MIXED function of SAS v.9.4 software (SAS Institute Inc., Cary, NC, USA). The normality of the data was assessed both before the analysis and for the resulting residuals after the analysis using PROC UNIVARIATE (Shapiro–Wilk test), followed by the QQ plot statement. In cases where the normality assumption was not met, data were transformed using $\log_{10}\left(x+1\right)$ . Residuals were subsequently re-evaluated for normality using the same methods. The model’s fixed effects included the level of nutrition, OCM treatment, and the interaction between these factors, with each individual heifer serving as the random effect. In the absence of significant interactions, the main effects of maternal nutrition and OCM treatment were presented. Means were separated using the PDIFF function of SAS (Tukey–Kramer adjustment) and all results were reported as least squares means (LSMEANS) and standard error of the mean (SEM). After analysis, the LSMEANS and SEM were back transformed to the original scale. The largest SEM is reported. All plots were generated using ggplot2 v.3.4.1 in R Studio v.4.2.2. Statistical significance was set at a *p*-value ≤0.05, and tendencies were reported for 0.05 < *p* ≤ 0.1.

## 3 Results

### 3.1 Genes expression in fetal tissue

**Figure 1:**
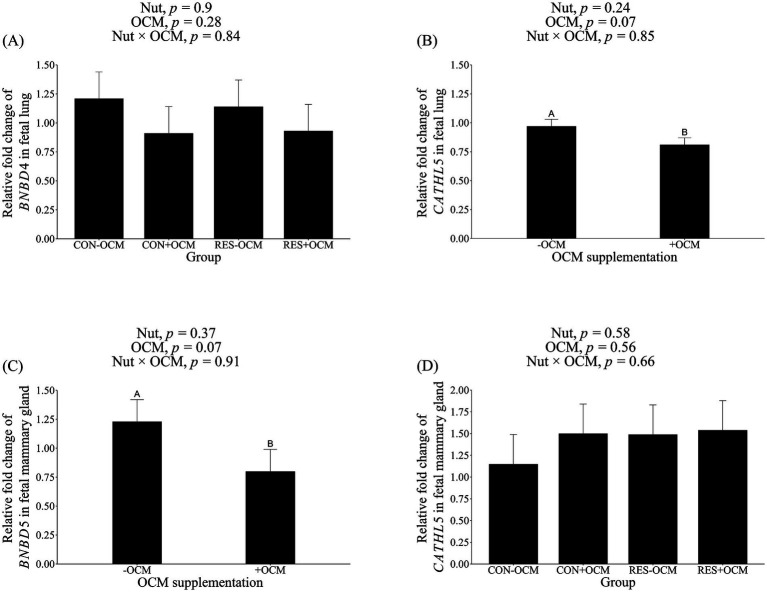
mRNA expression level of antimicrobial peptides in fetal tissues. **(A)** Bovine neutrophil β-defensin4 (*BNBD4*) in lung; **(B)** cathelicidin5 (*CATHL5*) in lung; **(C)** *CATHL5* in mammary gland; **(D)** bovine neutrophil β-defensin5 (*BNBD5*) in mammary gland. Data presented as a 2^-ΔΔCT^-fold change normalized to *GAPDH*, *ACTB*, and *HPRT1* as reference genes, and using the CON − OCM group as control. Different uppercase letters denote trends (0.05 < *p* ≤ 0.1).

The mRNA expression of *BNBD4* in the fetal lung was not influenced by either the nutritional plane × OCM interaction or the main effect of treatment (*p* > 0.28; Figure 1A; Table 2); however, there was a tendency (*p* = 0.07) for OCM supplementation to affect the mRNA expression of *CATHL5*, with expression being 16.5% lower in the +OCM groups (0.81 ± 0.06 relative fold) compared to the −OCM groups (0.97 ± 0.06 relative fold) (Figure 1B; Table 2).

**Table 2:** The effect of maternal nutritional levels [Nut; control (CON) or restricted (RES)]^1^ and supplementation with one-carbon metabolites [OCM; with OCM (+OCM) or without (−OCM)]^2^ from day 0 to 63 of gestation on mRNA expression of antimicrobial peptides measured in fetal lung and mammary gland.

				Supplementation					*p*-values		
Tissue	Gene^3^	Nut		−OCM	+OCM	SEM^4^	Nut^5^	SEM^6^	Nut	OCM	Nut × OCM
Lung	*BNBD4*	CON		1.21	0.91	0.23	1.06	0.15	0.90	0.28	0.84
		RES		1.14	0.93		1.03				
			OCM^7^	1.18	0.92						
			SEM^8^	0.16							
	*CATHL5*	CON		1.01	0.87	0.08	0.94	0.05	0.24	**0.07**	0.85
		RES		0.92	0.75		0.84				
			OCM	0.97^G^	0.81^H^						
			SEM	0.06							
Mammary gland	*BNBD5*	CON		1.44	0.81	0.27	1.12	0.19	0.37	**0.07**	0.91
		RES		1.03	0.78		0.90				
			OCM	1.23^G^	0.80^H^						
			SEM	0.19							
	*CATHL5*	CON		1.15	1.50	0.34	1.33	0.24	0.58	0.56	0.66
		RES		1.49	1.54		1.52				
			OCM	1.32	1.52						
			SEM	0.24							

The data are presented as the least square mean and standard error of the mean (SEM). ^1^CON (0.45 kg•heifer^−1^•day^−1^); RES = (−0.23 kg•heifer^−1^•day^−1^). ^2^+OCM = (with one-carbon metabolites supplementation); −OCM = (without one-carbon metabolites supplementation). ^3^*BNBD4* = Bovine neutrophil β-defensin4; *CATHLC5* = Cathelicidin5; *BNBD5* = Bovine neutrophil β-defensin5. ^4^Average SEM for nutritional levels × OCM supplementation interaction [CON – OCM (*n* = 7); CON + OCM (*n* = 8); RES – OCM (*n* = 7); RES + OCM (*n* = 7)]. ^5^Main effect of nutritional levels. ^6^SEM for Nut. ^7^Main effect of OCM supplementation. ^8^SEM for OCM supplementation. ^G,H^ Means within a row marked with uppercase letters denote trends (0.05 < *p* ≤ 0.1) for main effects.

In the fetal mammary gland, the mRNA expression of *BNBD5* tended (*p* = 0.07) to decrease by 35% in the +OCM groups (0.80 ± 0.19 relative fold) compared to the −OCM groups (1.23 ± 0.19 relative fold; Figure 1C; Table 2). No differences were observed in the mRNA expression of *CATHL5* among the groups (*p* > 0.1; Figure 1D; Table 2).

### 3.2 Genes expression in maternal tissue

**Figure 2:**
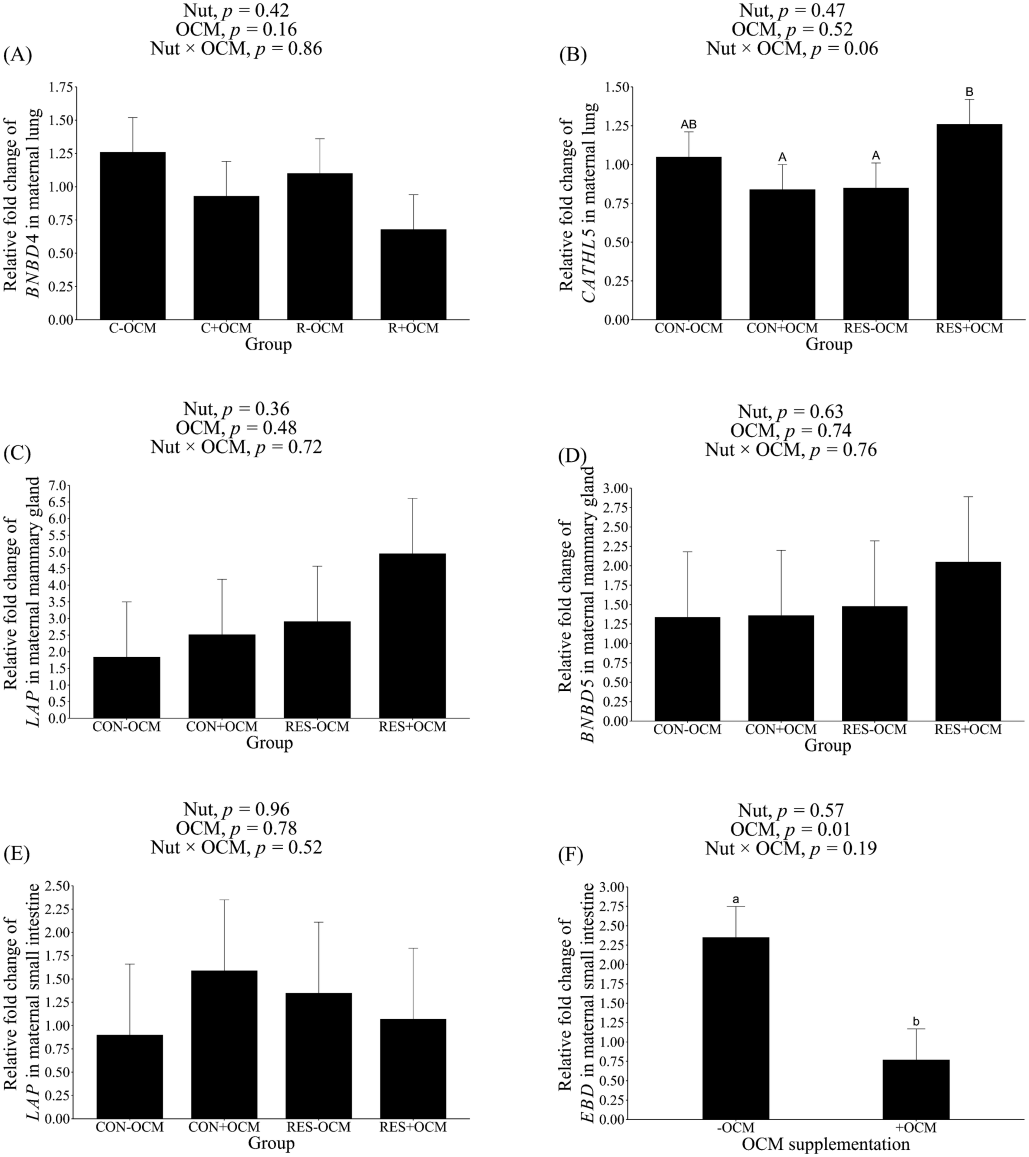
mRNA expression level of antimicrobial peptides in maternal tissues. **(A)** Bovine neutrophil β-defensin4 (*BNBD4*) in lung; **(B)** cathelicidin5 (*CATHL5*) in lung; **(C)** lingual antimicrobial peptide (*LAP*) in mammary gland; **(D)** bovine neutrophil β-defensin5 (*BNBD5*) in mammary gland; **(E)** *LAP* in small intestine; enteric β-defensin (*EBD*) in small intestine. Data presented as a 2^-ΔΔCT^-fold change normalized to *GAPDH*, *ACTB*, and *HPRT1* as reference genes, and using the CON − OCM group as control. Different lowercase letters indicate significant differences (*p* ≤ 0.05), while uppercase letters denote trends (0.05 < *p* ≤ 0.1).

In the maternal lung, the mRNA expression of *BNBD4* did not show differences among the groups (*p* > 0.1; Figure 2A; Table 3). Despite this, there was a tendency (*p* = 0.06) for an interaction for *CATHL5* mRNA expression, with the RES + OCM group having higher expression compared to the CON + OCM (*p* = 0.07) and RES − OCM (*p* = 0.08) groups (Figure 2B; Table 3).

**Table 3:** The effect of maternal nutritional levels [Nut; control (CON) or restricted (RES)]^1^ and supplementation with one-carbon metabolites [OCM; with OCM (+OCM) or without (−OCM)]^2^ from day 0 to 63 of gestation on mRNA expression of antimicrobial peptides measured in maternal lung, mammary gland, and small intestine.

				Supplementation					*p*-values		
Tissue	Gene^3^	Nut		−OCM	+OCM	SEM^4^	Nut^5^	SEM^6^	Nut	OCM	Nut × OCM
Lung	*BNBD4*	CON		1.26	0.93	0.26	1.10	0.18	0.42	0.16	0.86
		RES		1.10	0.68		0.89				
			OCM^7^	1.18	0.81						
			SEM^8^	0.18							
	*CATHL5*	CON		1.05^AB^	0.84^A^	0.16	0.95	0.11	0.47	0.52	**0.06**
		RES		0.85^A^	1.26^B^		1.06				
			OCM	0.95	1.05						
			SEM	0.10							
Mammary gland	*BNBD5*	CON		1.34	1.36	0.84	1.35	0.65	0.63	0.74	0.76
		RES		1.48	2.05		1.77				
			OCM	1.41	1.71						
			SEM	0.65							
	*LAP*	CON		1.84	2.52	1.66	2.18	1.44	0.36	0.48	0.72
		RES		2.91	4.95		3.93				
			OCM	2.37	3.74						
			SEM	1.36							
Small intestine	*LAP*	CON		0.90	1.59	0.76	1.25	0.52	0.96	0.78	0.52
		RES		1.35	1.07		1.21				
			OCM	1.13	1.33						
			SEM	0.52							
	*EBD*	CON		1.90	0.91	0.57	1.41	0.40	0.57	**0.01**	0.19
		RES		2.80	0.62		1.70				
			OCM	2.35^g^	0.77^h^						
			SEM	0.40							

The data are presented as the least square mean and standard error of the mean (SEM). ^1^CON (0.45 kg•heifer^−1^•day^−1^); RES = (−0.23 kg•heifer^−1^•day^−1^). ^2^+OCM = (with one-carbon metabolites supplementation); −OCM = (without one-carbon metabolites supplementation). ^3^*BNBD4* = Bovine neutrophil β-defensin4; *CATHLC5* = Cathelicidin5; *BNBD5* = Bovine neutrophil β-defensin5; *LAP* = Lingual antimicrobial peptide; *EBD* = enteric β-defensin. ^4^Average SEM for nutritional levels × OCM supplementation interaction [CON **−** OCM (n = 7); CON + OCM (*n* = 8); RES **−** OCM (*n* = 7); RES + OCM (*n* = 7)]. ^5^Main effect of nutritional levels. ^6^SEM for Nut. ^7^Main effect of OCM supplementation. ^8^ SEM for OCM supplementation. ^A,B^Means within a gene marked with uppercase letters denote trends (0.05 < *p* ≤ 0.10) for interactive effects. ^g,h^Means within a row marked with lowercase letters indicate significant differences (*p* ≤ 0.05) for main effects.

The mRNA expression of *BNBD5* and *LAP* was assessed in the maternal mammary gland. No differences (*p* > 0.1) in the expression of these genes were observed (Figures 2C,D; Table 3).

In the maternal small intestine, the mRNA levels of *LAP* did not differ among the groups (*p* > 0.1; Figure 2E; Table 3). However, the expression of *EBD* was affected by OCM supplementation (*p* = 0.01), with the +OCM groups (0.77 ± 0.4 relative fold) showing a 67% lower expression compared to the −OCM groups (2.35 ± 0.4 relative fold; Figure 2F; Table 3).

## 4 Discussion

This study investigated the effects of maternal nutrient restriction during early gestation, with or without OCM supplementation, on the expression of AMP in the fetal and maternal lung, small intestine, and mammary gland tissues of beef cattle. Antimicrobial peptides play a crucial role in innate immunity, acting as a first line of defense against pathogens. To our knowledge, this is the first study to examine the impact of restricted maternal nutrition and OCM supplementation on AMP expression.

We aimed to assess the mRNA expression of several selected AMPs in bovine fetal and maternal tissues, based on previous research. Specifically, we chose representative members from three major AMP families—β-defensins, cathelicidins, and S100 proteins—that have been shown to be more highly expressed in these tissues compared to other family members. Most prior studies have been conducted *in vitro*, meaning that the influences of factors, such as the environment, have not been fully considered. Additionally, there is limited literature on AMP expression in bovine fetuses, which is critical for understanding the development of the innate immune system and how environmental factors might impact immunity across generations. To our knowledge, aside from studies on *TAP* in the lung (55) and EBD in the small intestine (56), no previous research has explored AMP expression in bovine fetal tissues.

To ensure the accuracy, reliability, and reproducibility of our RT-qPCR results, we validated primers and amplification efficiency within the CON − OCM group. Our findings revealed that *S100A7* and *TAP* did not fall within the acceptable amplification efficiency range, likely due to low expression levels, as they exhibited high Ct values at the highest cDNA concentrations. Thus, we deemed the mRNA expression of these genes to be below our limit of detection. This could be attributed to several factors. Previous *in vivo* studies have demonstrated variation in the expression of AMPs in a single tissue analyzed from several animals of the same age group in cattle (55) and sheep (57, 58), which can account for this discrepancy.

Furthermore, previous studies in humans (59) and sheep (57, 58) models have demonstrated the developmental regulation of AMPs expression from fetus to neonate, with defensins showing increased expression during gestation. For instance, *α*-defensins in human fetal Paneth cells were detectable only after the 19th–24th week (60). It has also been shown that certain AMPs predominant in adulthood were not detectable in the fetus, both in human (59) and bovine models (55). Our data indicated low or absent expression of certain AMPs, such as in the fetal small intestine, aligning with findings that developmental regulation might only become apparent closer to term. It has been observed that the expression of certain β-defensins is lower in preterm humans than in term neonates and adults (60).

The birth delivery method can influence AMPs gene expression. Data indicate increased AMPs synthesis during vaginal delivery due to labor stress (60). In bovine neonates, *LAP* expression in the lung was lower following elective cesarean section compared to transvaginal delivery (61). Since our samples were collected from fetuses at 161 days of gestation without labor stress, the absence of certain gene expressions is logical.

Tissue-specific, or more precisely tissue-region-dependent, AMPs expression is another factor. For example, *TAP* is restricted to airway tissue, with the majority of expression in the trachea in cattle (55). In our study, lung samples were collected from the cranioventral region, where respiratory pathogens typically impact, and *TAP* was either not expressed or lowly expressed. This finding suggests that *TAP* is not constitutively expressed in the lung tissue of either mature cattle or their fetuses. Additionally, Tetens et al. showed a high level of *S100A7* mRNA expression in the streak canal and the Rosette of Fürstenberg of the mammary gland; however, they could not detect *S100A7* mRNA expression in the gland cisternal epithelium and udder parenchyma of healthy cattle (62). In agreement, our study found that *S100A7* was either not expressed or lowly expressed in both fetal and maternal parenchyma of the mammary gland. Therefore, according to our findings and previous studies, it is conceivable that *S100A7* is not constitutively expressed in the parenchyma of the mammary gland of both fetus and dam.

Inducible expression in response to stimuli is a characteristic of certain AMPs. In bovine studies, the inducible properties of β-defensins in the lung (63), mammary gland (64) and small intestine (56) have been properly shown, where AMPs expression was either low or nonexistent in the absence of stimuli. For example, *LAP* mRNA was significantly upregulated in the intestinal mucosa during chronic *Mycobacterium paratuberculosis* infection and in the bronchiolar epithelium during acute *Pasteurella haemolytica* infection, with low or absent signals in non-infected tissues (63). The absence of such stimuli in our study could explain the low or absent expression of certain AMPs.

### 4.1 Impact of maternal nutritional restriction on expression of AMP

Maternal undernutrition prior to parturition has been shown to interfere with the innate immune system of the ovine fetus and offspring (4, 65). However, to our knowledge, no studies have explored the effects of restricted maternal nutrition during pregnancy on the bovine fetal expression of AMP. Our data indicate that restricted maternal nutrition did not significantly affect the expression of AMP, neither in the fetus nor in the dams.

Several potential reasons may explain why the restricted nutritional plane did not independently result in significant changes in AMP expression. First, the severity and duration of the nutrient restriction imposed in this study may not have been sufficient to elicit significant changes in AMP expression. Although the extent of restricted nutrition was sufficient to assess impacts on gene expression associated with tissue metabolism, accretion, and function in our previous studies (66, 67), it may not have been severe or prolonged enough to affect AMP expression significantly.

Second, epigenetic adaptations might have occurred to maintain baseline AMP expression levels, potentially as a protective measure against nutrient restriction. It has been pointed out that the impact of prenatal events, such as undernutrition, on the immune system is not uniform. These events may lead to differential investments in various subsystems of the immune system, where some aspects might be downregulated or compromised, while others receive increased investment or remain unaffected (68, 69). In this context, it is possible that the body prioritized the maintenance of AMP expression over other immune functions in response to the prenatal nutritional stress experienced in our study. This differential investment could be an adaptive response, as it proposes that the immune system may shift its investment away from more energetically expensive specific immune defenses (like those involving specific antibodies and adaptive immune responses) and towards less costly nonspecific defenses (such as innate immune defenses) (68). This shift could help conserve resources while still providing adequate protection. It has been shown that undernutrition in ewes can upregulate specific genes, such as Cholesterol 25-Hydroxylase and MHC Class I Polypeptide-Related Sequence B, that modulate the innate immune response and inhibit the actions of receptors involved in cytotoxic activities, potentially beneficial for immunotolerance at the embryo-maternal interface (70).

Another factor is the variability in tissue response to stimuli. The AMP are constitutively expressed but can also be induced/increased in response to stimuli such as infection (17). Studies have shown that different energy levels during gestation do not affect the mRNA abundance of innate immune-related genes in weaned piglets under basal conditions; however, when challenged with lipopolysaccharide, the immune response was significantly higher in those born to dams under low-energy diets (71). Therefore, the lack of significant changes in AMP expression in the current study may be due to the absence of an immunological challenge. Hence, it is suggested that evaluating the expression of AMP in both dams and fetal tissues in response to different immunological challenges could provide further insights into the potential effects of maternal nutrition on the inducible expression of these innate immune effectors.

Additionally, maternal organisms may have evolved compensatory mechanisms to mitigate the effects of nutrient restriction on the developing fetus. These mechanisms could include alterations in nutrient partitioning, placental adaptation, or changes in maternal metabolism to ensure the provision of essential nutrients to the fetus, thereby minimizing the impact on fetal development (72, 73), including AMP expression.

Furthermore, sex-specific effects could play a role. Although the effects of sex were not explored in this study, there is strong evidence supporting sexual dimorphism in the innate immune system. Females tend to have a more effective and protective innate immune response, leading to better outcomes in the face of infections and immune challenges (9, 74). The mechanisms behind these observations may include evolutionary adaptations for higher reproductive potential (75) and hormonal differences between males and females (74).

### 4.2 Impact of OCM supplementation on expression of AMP

The supplementation of OCM during early gestation influenced the expression of AMP in both fetal and maternal tissues, indicating a modulatory role of these metabolites on the innate immune system.

In the fetal lung and mammary gland, the mRNA expression of *CATHL5* and *BNBD5*, respectively, tended to decrease in the OCM-supplemented groups compared to the non-supplemented groups. Similarly, in the maternal small intestine, the mRNA levels of *EBD* were significantly lower in the OCM-supplemented groups. These findings demonstrate that the expression of AMP was suppressed by OCM supplementation.

One-carbon metabolism and its metabolites are essential for the proper regulation of immune function through epigenetic modifications and metabolic reprogramming, ensuring that immune cells can respond effectively to infections and stress (13, 14). For instance, a study by Sinclair et al. (9) demonstrated that a maternal diet low in B vitamins and methionine can lead to altered immune responses in ovine offspring, indicating potential negative impacts on the immune system (9). Although our results showed a decrease in the mRNA expression of AMP in response to OCM supplementation, this does not necessarily contradict previous findings. It is possible that the improved overall condition of the OCM-supplemented fetuses reduced the need for immune responses, thereby influencing the expression of these immune-related genes. Further research is warranted to evaluate the activation and function of AMP in response to OCM supplementation.

It is widely acknowledged that the expression of AMP increases during an inflammatory response (76, 77). The nuclear factor-kappa B (NF-κB) through toll-like receptor (TLR)-NF-κB pathway, and the mitogen-activated protein kinase (MAPK) signaling pathway play crucial roles in regulating the expression of AMP (17, 78). On the other hand, a deficiency in one-carbon metabolites has been shown to elevate homocysteine levels, leading to increased inflammation. In agreement, our previous research has shown that nutrient restriction in beef heifers can result in reduced concentrations of methionine in allantoic fluid and increased levels of homocysteine in maternal serum during early gestation (16). Conversely, OCM reduces inflammation in two ways. First, it increases levels of S-adenosylmethionine (SAM) while reducing S-adenosylhomocysteine (SAH). The ratio between these compounds (SAM/SAH) regulates most methyltransferases (79–82). Second, when SAM levels rise, they help decrease inflammation by reducing NF-κB production and suppressing both the NF-κB and MAPK pathways. This leads to lower levels of inflammatory markers (80, 83, 84). Therefore, it is likely that OCM supplementation attenuated AMP expression by exerting its anti-inflammatory properties through the suppression of the NF-κB and MAPK pathways. Future studies should evaluate these pathways in the context of OCM supplementation to further elucidate their role in regulating AMP expression.

## 5 Conclusion

In conclusion, our study provides valuable insights into the effects of maternal nutrient restriction and OCM supplementation during early gestation on the mRNA expression of AMP in fetal and maternal lung, small intestine, and mammary gland tissues of beef cattle. We evaluated some AMP genes in fetal tissues for the first time, which is crucial for understanding the development of the innate immune system in cattle. Additionally, our findings revealed that restricted maternal nutrition alone did not significantly alter AMP expression; however, OCM supplementation led to a decrease in the mRNA expression of *CATHL5* and *BNBD5* in fetal lung and mammary gland tissues, respectively, and a significant reduction in *EBD* expression in the maternal small intestine. Further research is warranted to elucidate the mechanisms by which OCM modulate AMP expression, potentially through exerting anti-inflammatory effects by suppressing the NF-κB and MAPK pathways.

## Data Availability

The original contributions presented in the study are included in the article/Supplementary material, further inquiries can be directed to the corresponding author.
