# Supplementary material for: Influence of maternal nutrition and one-carbon metabolites supplementation on bovine antimicrobial peptides in fetal and maternal tissues
Source: Front Vet Sci. 2024 Dec 10;11:1505427. doi: 10.3389/fvets.2024.1505427 (PMC11666495; doi:10.3389/fvets.2024.1505427)
Supplement: Supplementary file 1 [file Data_Sheet_1.PDF]

Table S1. Primer validation and amplification efficiency for antimicrobial peptide genes in fetal lung, small intestine, and mammary gland using a six-point relative standard curve through five 4-fold serial dilutions from 50 ng to 0.0488 ng cDNA per reaction. All runs performed in triplicate.

Tissue	Gene[1]	Source[2]	Slope[3]	(R^2)[4]	Efficiency[5]	Comment
Lung	*LAP*	First	-2.702	0.904	134.475	
		Second	-2.613	0.933	141.405	
	TAP	First	-	-	-	No amplification
		Second	-	-	-	Amplification too late
	BNBD4	First	-3.57	**0.995**	**90.606**	
		Second	-2.897	0.981	121.379	
	CATHL5	First	-	-	-	Amplification too late; high Ct values
		Second	-3.08	**0.987**	**110.211**	
	GAPDH		-3.498	0.996	93.139	
	ACTB		-3.538	0.995	91.724	
	HPRT1		-3.403	0.994	96.709	
Small Intestine	*LAP*	First	-3.53	0.93	91.993	
		Second	-2.544	0.968	147.237	Amplification too late
	TAP	First	-1.407	0.535	413.488	Amplification too late; high Ct values
		Second	-0.98	0.168	947.862	Amplification too late
	EBD	First	-0.651	0.325	33,341	
		Second	-	-	-	No amplification
	CATHL5	First	-1.946	0.679	226.553	Amplification too late; high Ct values
		Second	-3.968	0.972	78.668	
	ACTB		-3.511	0.994	92.687	
Mammary gland	*LAP*	First	-	-	-	No amplification
		Second	-3.22	0.973	104.428	
	BNBD5	First	-3.362	**0.999**	**98.35**	
		Second	-3.257	0.994	102.796	
	CATHL5	First	-3.27	**0.994**	**102.196**	
		Second	-3.05	0.986	112.76	
	S100A7	First	-	-	-	No amplification
		Second	-3.765	0.967	84.33	
	GAPDH		-3.487	0.999	93.545	

[1] *LAP* = lingual antimicrobial peptide, *TAP* = tracheal antimicrobial peptide, *EBD* = enteric β-defensin, *BNBD4* = bovine neutrophil β-defensin4, *BNBD5* = bovine neutrophil β-defensin5, *CATHL5* = cathelicidin5, *S100A7* = S100 calcium binding protein A7. *GAPDH* = Glyceraldehyde-3-phosphate dehydrogenase. *ACTB* = Actin beta. *HPRT1* = Hypoxanthine-guanine phosphoribosyltransferase1. [2] The primers designed in this study are referred to as (first primer), while the primers sourced from previous literature are referred to as (second primer) for each gene. [3] Slope of the standard curve. [4] R^2 stands for the coefficient of determination of the standard curve. [5] Efficiency is calculated as $[10^{-1/slope}-1]$.

Table S2. Primer validation and amplification efficiency for antimicrobial peptide genes in maternal lung, small intestine, and mammary gland using a six-point relative standard curve through five 4-fold serial dilutions from 50 ng to 0.0488 ng cDNA per reaction. All runs performed in triplicate.

Tissue	Gene[1]	Source[2]	Slope[3]	(R^2)[4]	Efficiency[5]	Comment
Lung	*LAP*	First	-2.875	0.984	122.78	
		Second	-2.744	0.931	131.42	
	TAP	First	-3.915	0.997	80.056	
		Second	-3.584	0.807	90.105	
	BNBD4	First	-3.517	**0.99**	**92.45**	
		Second	-	-	-	No amplification
	CATHL5	First	-2.404	0.278	160.61	
		Second	-3.321	**0.992**	**100.022**	
	GAPDH		-3.48	0.999	93.78	
	ACTB		-3.35	0.996	98.858	
	HPRT1		-3.093	0.997	110.54	
Small Intestine	*LAP*	First	-3.156	**0.991**	**107.43**	
		Second	-2.911	0.967	120.54	
	TAP	First	-3.85	0.986	81.856	
		Second	-2.718	0.831	133.316	
	EBD	First	-	-	-	No amplification
		Second	-.3515	**0.996**	**92.518**	
	CATHL5	First	-	-	-	No amplification
		Second	-1.021	0.294	853.323	High Ct values
	GAPDH		-3.542	0.998	91.58	
	ACTB		-3.441	0.999	95.245	
	HPRT1		-3.292	0.992	101.28	
Mammary gland	*LAP*	First	-	-	-	No amplification
		Second	-3.184	**0.993**	**106.09**	
	BNBD5	First	-3.466	**0.99**	**94.324**	
		Second	-	-	-	No amplification
	CATHL5	First	-3.364	0.963	98.266	
		Second	-2.825	0.994	125.934	
	S100A7	First	4.536	0.194	-39.808	
		Second	-2.733	0.959	132.226	
	GAPDH		-3.421	0.997	96.014	
	ACTB		-3.445	0.998	95.101	
	HPRT1		-3.513	0.99	92.61	

[1] *LAP* = lingual antimicrobial peptide, *TAP* = tracheal antimicrobial peptide, *EBD* = enteric β-defensin, *BNBD4* = bovine neutrophil β-defensin4, *BNBD5* = bovine neutrophil β-defensin5, *CATHL5* = cathelicidin5, *S100A7* = S100 calcium binding protein A7. *GAPDH* = Glyceraldehyde-3-phosphate dehydrogenase. *ACTB* = Actin beta. *HPRT1* = Hypoxanthine-guanine phosphoribosyltransferase1. [2] The primers designed in this study are referred to as (first primer), while the primers sourced from previous literature are referred to as (second primer) for each gene. [3] Slope of the standard curve. [4] R^2 stands for the coefficient of determination of the standard curve. [5] Efficiency is calculated as [$10^{-1/slope}$-1].
